# New York Letter

**Published:** 1894-04

**Authors:** Wm. L. Russell

**Affiliations:** 101 West 93d St.; New York


### *CORRESPONDENCE.*

---

### OUR NEW YORK LETTER.

New York, March 16, 1894.

The approaching International Medical Congress at Rome furnishes probably sufficient reason for the unusual departure of the Academy, at the meeting last evening, from pure scientific work to the consideration of a universal language for scientific men. It was the opinion of Dr. Jacobi that the subject should have been brought before the Congress itself, as Italian had now been added to the three official languages, and others might be expected when other congresses were held in different countries. The result of this he thought would be the disappearance of these congresses, as nothing could be more unsatisfactory than the present confusion of tongues that prevailed. It was quite customary for all unfamiliar with the language in which an address was being delivered, to leave the room by the hundred. He thought, however, that any language must be learned in childhood in order to be made practicable for the purpose suggested. He had know children who could speak three languages with ease.

The paper of the evening was read by Dr. Achilles Rose, and consisted of an elaborate discussion of the claims of the Greek for adoption as an international language for scholars. He said that no one who had visited an International Medical Congress could fail to have noticed the confusion of tongues and the desirability of a common medium of communication.

The Philosophical Convention, in connection with the Paris Exposition, had set its face against Volapük, and there was no possibility of it ever becoming practicable. The Greek was not a dead language like the Latin, and yet the people who spoke it were sufficiently obscure to prevent the rivalry which formed an obstacle to the adoption of English, French or German. Greek was already studied in schools and colleges and all that was necessary was to change the methods of teaching it, now wofully deficient, and allow the student to acquire it as he did his mother tongue. It was true that modern Greek was not identical with the ancient, but the difference was no greater than that between the English of Chaucer and that of the present day. This could be easily determined by an examination of current Greek literature, of which there was considerable. He who was familiar with modern Greek would have very little difficulty with the ancient.

Dr. C. A. Leale believed that a universal language was not possible without the re-education of whole nations. He thought that the indications were that the day for Greek had passed, and that English was becoming the dominant tongue. This was shown by statistics revealing that in 1801, 12.7 per cent. of the people of the world spoke English, 19 per cent. French, and 18.7 per cent. German; while in 1890, 27.7 per cent. spoke English, against 12.7 per cent. speaking French, and 18.7 per cent. speaking German.

In connection with his remarks, he told an anecdote of the late Dr. Peaslee which is well worth repeating. An eminent English scientist was invited to address the Academy on the subject of his life-work and writings. He delivered a most elaborate address, but much to the consternation of Dr. Leale, whose duty as Secretary required him to take notes, he punctuated his remarks with Greek words, phrases, and sentences, sometimes speaking for a considerable period in that language. After the applause which followed his conclusion had subsided, Dr. Peaslee, who had been apparently asleep during the address, aroused himself, and rising to his feet corrected the quotations of the learned Englishman, rehearsing many of the passages and referring them to their proper authors. Dr. Leale had never heard him quote Greek in the Academy before, and the present occasion would probably prevent at least one visiting confrère from doing it again.

Dr. Wm. H. Thomson did not believe an international language for scholars to be practicable until evolution in the system of education, and co-operation of the educators of the world had been secured. The present method of teaching languages in the schools was by the eye, the result of which was that they were inadequately learned for practical purposes. In order to be able to use a language with facility, one must be able to think in it. And in order to do that it must be learned by the ear.

If an international language should become practicable, he thought Greak best of all. It was a living language, and most musical to hear. Best of all it abounded in partitives. Language could be divided into nouns, the names of things, verbs, the names of events, and partitives which connected the two. English was particularly deficient in the latter, and was becoming more so, on account of the abandonment of such words as whereby, hereof, bystander, onlooker, etc. Scientific men already went to the Greek for many words and parts of words.

During the last century French was the universal language of polite society in Europe, and it was not until 1828 that it ceased to be the language of diplomatic usage. There was great need of a common language for scientific men, and it might perhaps lead to the adoption of better methods of teaching in the schools.

Dr. S. S. Burt believed that the language spoken most prevalently must be that of the dominant nations; for this reason he believed that English promised to prevail in the end.

The President, Dr. Roosa, after referring to the appropriateness of the discussion, said that International Medical Congresses were dismal failures. He could hardly believe that English could become sufficiently pre-eminent, as long as other great nations existed. The methods of teaching languages, both ancient and modern, which existed at the great universities, were so deficient and useless for practical purposes, that he wondered that any civilized nation could perpetuate them.

### BRONCHITIS IN CHILDREN.

This was the subject at the last meeting of the Section on Pediatrics. Dr. C. E. Kerley read a paper describing the treatment pursued at the New York Infant Asylum for children under one year old. The temperature of the ward was kept at 70° to 72°, and every twelve hours the windows were opened, and complete change of the air secured. Loose garments of flannel were worn, and the woollen band was discarded, as it restricted respiration. If the case were of long standing, or the child much debilitated, an oiled silk jacket was applied. The position of the child was changed frequently, especially if it lain much on its back. A lukewarm salt-water bath was given morning and evening.

If there was pronounced dyspnœa and a hard cough, a mustard bath at 100° to 110°, or a mustard pack, was given not oftener than twice daily. If the small bronchi were involved the steam spray for fifteen minutes every hour, or sometimes continuously for several hours, was employed, a rubber sheet being applied to protect the clothing.

As a local measure, counter-irritation gave the best results. This was obtained by the use of oil of turpentine with camphorated oil, or the latter alone, rubbed in until the skin was reddened. When a decided effect was necessary, mustard paper four or five inches by twelve, was applied for one to three minutes. It made the child cry, which was desirable. In other cases a mustard paste in the proportion of one part of mustard to five of flour was applied between cloths twice daily. The medicinal treatment consisted in the use of powdered ipecac, and tartar emetic if the case was seen early. Muriate of ammonia was then given. An emetic was not often required. If it were wine of ipecac, fifteen minims, every fifteen minutes, were given. If the case tended to chronicity, cod-liver oil was given for a long period. If the condition of the heart required it, alcohol was used. In some instances, when it seemed indicated on account of the condition of the bowels, three to five drops of castor oil were given every two hours for several days.

Dr. H. Koplik reviewed the treatment for children over one year old. He said that in a healthy child recovery was the natural outcome. A mild opiate was sometimes necessary in the acute stage to allay cough. Afterwards all measures that reduced the vitality should be avoided. He used no emetics and no aconite. He also avoided baths, as exposure might result in aggravation of the disorder. The medicines employed were paregoric, not more than four minims every three or two hours, in a child under six, and syrup of ipecac. During convalescence, strychnia, gr. $\frac{1}{500}$, was useful, and terebene, half a minim to two minims in tragacanth emulsion or on sugar. He did not believe poulticing, expectorants, quinine, whisky, or brandy, digitalis, or the use of an oiled silk jacket, to be indicated or desirable in these cases.

In another class of cases in which the constitution was undermined by rachitis, syphilis, etc., there was a tendency for the disease to become subacute or chronic. Here cod-liver oil and phosphorus were the main stays. Potassium iodide could be added in the syphilitic cases. When bronchitis was a complication of other diseases, the treatment was often far from simple, and heart stimulants and antipyretic measures might be required.

In the discussion, Dr. Wm. H. Thomson said that he doubted if bronchitis was often primary. It was generally the result of influences affecting the skin. He had found that the wearing of a towel around the neck at night was very useful in chronic and protracted cases. Children often perspired freely about the head and neck at night, and for these cold sponging and the towel protector were invaluable. The most important factor in acute bronchitis in young children, was their natural muscular weakness, which lessened the power of expectoration from the pharynx, allowing mucus to collect about the epiglottis. This was one reason why an emetic was often useful in these cases, and great care should be taken to remove all the mucus within reach. When the small bronchi were filling, nothing was more useful than the constant swallowing of hot drink, this measure having been demonstrated to increase the heart power. He believed mustard somewhat dangerous for use on the skin of a child, as he had seen two cases in which gangrene and death had resulted. He employed capsicum half a drachm to a drachm in a pint of boiling water, in which cloths were soaked and wrapped around the chest. Muriate of ammonium was well borne by children a year old, if given in milk every hour or two. As much as three grains at a dose could be used.

Dr. S. Baruch preferred to prevent bronchitis by enhancing the child's powers of resistance. This could be done by means of a process of hardening by cool and tepid bathing. The method to pursued was to begin with water at 80° which should be applied to the child by the hand. The temperature of the water should be reduced 2° daily, but should never be under 60° for a child under five years old. If the disease were already present in an acute form, he began treatment by administering two to five grains of calomel. This was followed by measures to dilate the surface vessels, thus relieving the heart. He applied a jacket made of cotton wadding. He used no expectorants, but gave baths, beginning with a temperature of 95° and reducing to 80°, with friction. This served to relieve the heart by promoting the cutaneous circulation.

The Chairman, Dr. Winters, believed that prolonged poulticing did harm by weakening the muscles of respiration. He regarded nux vomica as a valuable stimulant to these muscles.

### PHYSIOLOGY OF DEGLUTITION.

An interesting contribution to this subject was presented at the last meeting of the Section on Neurology, by Dr. S. J. Metzler. He said that experiments made on himself had shown that the act was much too rapid to be depended on in peristalsis. He had found that it was due, in a measure, to the mouth force exerted by means of the mylo-hyoid muscle, which acted in a manner similar to a bulb syringe. The esophagus could be divided anatomically into three sections. The first contained striated muscle, the second contained mixed muscular fibres, and in the third the fibres were plain. When the food was dry, and when, as in the rabbit, all the muscle was unstriated, swallowing was very slow. If a stethoscope were placed to the left of the ensiform cartilage, six or seven seconds after the act of deglutition, a prolonged sound was heard, which was due to the contraction of the last section of the esophagus. Sometimes a sound was heard immediately after the act of deglutition, but this was squirting in character. When a number of acts of deglutition were made to follow one another in rapid succession, the prolonged sound was heard only after the last. The same sounds could be heard behind from the fifth to the ninth dorsal vertebræ.

It had been noticed that a single act of deglutition accelerated the pulse rates by its inhibiting effect on the vagus. The rate rose from seventy-two to a hundred per minutes. Respiration, too, was inhibited by drinking, even in tracheotomized animals. Repeated acts of swallowing were helpful in fainting, singultus and in dyspnœa, a short sharp descent of the diaphragm occurring at each act.

Dr. Max Einhom believed auscultation for the sounds referred to was of value in diagnosing stricture of the esophagus.

Dr. Wm. H. Thomson referred, also, to the value of the effect of deglutition on the heart in cases of bronchitis in children.

Dr. Mary Putnam Jacobi, chairman of the section, thought the point might be applied in the management of children prematurely born. She could also see the bearing it might have as an explanation of the fact that the presence of cardiac disease was a contra-indication to lavage of the stomach.

Dr. Metzler said in closing, that he hardly thought that lavage was likely to cause injury in cardiac diseases, usless from pressure caused by the full stomach.

WM. L. RUSSELL.

101 *West 93d St.*

---

### FOR ACUTE RHEUMATISM.

℞ Sodii salicyl. ........ ʒ vi.

Glycerinæ ........ ʒ i.

Aquæ cinnamoni ........ ℥ vi.

M. S.—A teaspoonful every two hours until tinnitus aurium is produced, then every four to six hours until acute symptoms have abated, then give—

℞ Sodii bicarbonatis ........ ʒ iv vj.

In pulv. No. xij divide.

Sig.—A powder in half a glass of water until the urine is alkaline to test paper. If patient is anemic, begin on soda at once, omitting the salicylate, and give cod-liver oil and iron from the start.

—*A. L. Loomis, Bellevue Hospt., N. Y.*

---

### RHEUMATIC SORE-THROAT.

Dr. Fletcher Ingals, as a topical application, uses the following pigment:

℞ Morphin. sulph ........ gr. iv.
Ac. carbolic,
Ac. tannic ........ aa. gr. xxx.
Glycerini,
Aquæ dest ........ aa. ʒ iv.

M. S.—Apply locally.

—*Medical Bulletin.*

